# The need for acute assessments in home healthcare - Swedish registered nurses’ experiences

**DOI:** 10.1080/17482631.2024.2373541

**Published:** 2024-06-27

**Authors:** Jessica Westerholm, Lena-Karin Gustafsson, Mirkka Söderman

**Affiliations:** a Tranemo Municipality, Sweden; b Division of Caring Science, School of Health, Care and Social Welfare, Mälardalen University, Eskilstuna, Sweden

**Keywords:** Acute assessments, decision support system, home healthcare, patient safety, qualitative content analysis, qualitative survey

## Abstract

**Purpose:**

The study aims to describe Swedish RNs’ experiences of acute assessments at home. More patients with complex nursing needs are cared for at home due to an ageing population. Registered nurses (RNs) who work with home healthcare need a broad medical competence and clinical experience alongside adapted decision support systems for maintaining patient safety in acute assessments within home healthcare.

**Methods:**

A content analysis of qualitative survey data from RNs (*n* = 19) working within home healthcare in Sweden.

**Results:**

There were challenges in the acute assessments at home due to a lack of competence since several of the RNs did not have much experience working as an RN in home healthcare. Important information was missing about the patients, such as access to medical records due to organizational challenges and limited access to equipment and materials. The RNs needed support in the form of cooperation with a physician, support from colleagues, and a decision support system.

**Conclusion:**

To increase the possibility of patient-safe assessments at home, skills development, collegial support, and an adapted decision support system are needed. Collaboration with primary healthcare, on-call physicians, and nursing staff, and having the opportunity to consult with someone also provide security in acute assessments.

## Introduction

As populations age, home healthcare appears to be on the increase throughout Europe. At the same time, long-term disease states are more common, which in turn increases the risk of suffering from acute illness (Lee et al., 2017). Hospitals are mostly the standard of care for acute illness, but inpatient care is expensive and demands high competence resources (Levine et al., 2020). Hospitalization in acute care units poses a significant risk to the functional independence of older adults. This risk is unchanged despite shorter lengths of stay at the hospital based acute unit (Loyd et al., 2020). The outcoming knowledge after the COVID-19 pandemic has supported the accelerated opportunities to keep frail older adults safely in their homes despite them having medically complex needs (Franzosa et al., 2021; Moretti et al., 2020).

Home healthcare is a phenomenon that has changed over time and enables patients with complex nursing needs to be cared for at home. This, along with ongoing technological developments, mean short treatment times and even quick discharges from hospital (Fjørtoft et al., 2021). In Sweden, home healthcare is provided to varying patient groups in different forms (Swedish Agency for Health and Care Services Analysis, 2021). The municipalities are responsible for care efforts including home healthcare and rehabilitation. The RN has a central and coordinating role when it comes to care interventions and has the authority to delegate certain care interventions to nursing staff within home care, i.e., assistant nurses and care assistants providing basic healthcare, social care, and home service. The RN accounts for most of the medical expertise, but physicians are also connected to home healthcare. The healthcare can consist of caring for existing health care needs, curative care, end-of-life care as well as caring for chronic or acute disease states. Even if patients with a broad spectrum of diagnoses are treated at home and even if they are provided with technology from hospital care, the conditions for home healthcare are not the same as in hospitals. The hospitals have access to assistant nurses specifically trained to take care of the care of these seriously ill patients and to act in emergency situations. In addition, RNs have better opportunities to supervise these assistant nurses in nursing. Registered nurses (RNs) in home healthcare face many challenges to ensure patient-safe care, as care becomes increasingly advanced and they are responsible for many critically ill patients (Andersson et al., 2017; Pavloff & Labrecque, 2021). For example, acute infections being assessed by RNs are common, although there is often a lack of routines for how these patients should be assessed or cared for at home, which can lead to patients not receiving the right treatment in time (Dowding et al., 2020). It is also common that RNs do not have access to essential information about the patient at home, and this may put the patient in danger (Dowding et al., 2020).

Pre-hospital decision support systems (DSS) are more common in the ambulance service than in home healthcare, although RNs in home healthcare are also faced with acute assessments. The RN’s role in home health care is autonomous, their assessments and clinical reasoning skills are critical to outcomes and recommendations for continued patient care (Rusli et al., 2022). Assessment aids used in assessments within home healthcare need to be adapted and tested for the patient group in order to be useful and patient safe (Jeppestøl et al., 2022). The Modified early warning score (MEWS) is a well-proven assessment system. However, when used to assess older patients in home healthcare, the utility of the tool is somewhat limited because it is not perceived as sufficiently adapted for home healthcare or for older patients. Other types of assessment instruments used in home healthcare, e.g., Abbey Pain Scale (Abbey et al., 2004) and Edmonton Symptom Assessment Scale (Hui & Bruera, 2017), are well proven in palliative care but are not fully adapted for an acute assessment of the frail older person cared for in homecare. There are promising DSS such as ViSam (Kihlgren et al., 2016), but it should be noted that while DSS are useful tools, they can never replace clinical judgement and nursing experience (Downey et al., 2017).

Regular skills development and more specialist training are needed, in addition to good cooperation between colleagues and other healthcare providers. Higher competence, i.e., knowledge and skills, of RNs’ in home healthcare, results in higher patient safety (Andersson et al., 2017; Pavloff & Labrecque, 2021), and factors such as experience, training, and knowledge of the patient increase the quality of the clinical assessment (Gray et al., 2018). Experienced RNs rely on their experience and intuition in a different way than those who are less experienced (Banning, 2008). Assessing and treating patients with many different symptoms is a common occurrence in all care at home, and advanced medical decisions often have to be made far from the hospital’s collective competence (Pavloff & Labrecque, 2021). Therefore, RNs need a care team with close interactions, flexibility and improvisation for high-quality home health care (Larsen et al., 2017).

Previous research on RNs’ experiences of acute assessments in the home has been studied mainly in the pre-hospital area (Sjölin et al., 2020; Suamchaiyaphum et al., 2024; Wästerhed et al., 2024). In home healthcare, acute assessments are often made by a lone RN who does not have access to the aids found in an ambulance or in inpatient care, which makes the complex assessment even more challenging. Incomplete assessments related to a lack of relevant competence or a DSS can lead to patient safety being compromised. Thus, more knowledge is needed about emergency care, even in health and medical care that is carried out in the home. The aim of this study was to describe Swedish RNs’ experiences of acute assessments in the home.

## Materials and methods

This survey with qualitative approach was conducted via a web-based questionnaire with open-ended responses and analysed according to Elo and Kyngäs (Elo & Kyngäs, 2008) and their description of inductive qualitative content analysis.

### Data collection

A web-based questionnaire in Swedish with open-ended qualitative questions was constructed in the “Microsoft Forms” Web programme (Appendix 1). A link to the survey including information about the study was sent via operation mangers, in two middle-sized municipalities in western Sweden, to all employed RNs working in municipal home healthcare, i.e., about 65 RNs. The choice of municipalities was based on geographical proximity to the first author. The RNs were invited to participate regardless of professional experience or degree of training as their tasks are largely the same within home healthcare. The questions were developed based on previous research, e.g., that acute assessments are often made by a lone RN and then we asked what support RNs felt they had. Also, through discussions with RNs working at one specific unit within home healthcare about which problems are common when it comes to acute assessments within home healthcare, and discussions between the authors. A pilot study was conducted, among four RNs working at a smaller unit for home healthcare, to test the questionnaire and the wording of some questions was revised. The questionnaire consisted of five background questions and 10 questions to answer the purpose of the study. The survey took 20–30 minutes to answer. Within three weeks from sending out the final survey link, responses were received from 19 RNs. The questionnaire was then closed when the material from the responses received was deemed to sufficiently fulfil the purpose of the study, i.e., when no new codes or headings emerged when reading through more questionnaires. All questions were answered by all participants, although not all answers were detailed.

### Participants

The 19 RNs who answered the survey were women aged 24 to 56 years (Table 1). They had varying lengths of experience working as RNs. The shortest experience was about 1 year and the longest over 30 years. The RNs’ experience in home healthcare ranged from six months to 28 years. Only four RNs had specialist nursing training after graduation, one in the care of older persons, two as public health and one as a midwife.

**Table 1.** Background information regarding registered nurses (*n* = 19).

	n
**Age**	
20–30	4
31–40	6
41–50	4
>50	5
**Gender**	
Female	19
Male	0
**Number of years as a RN**	
Up to 1 year	2
2–5 years	12
6–10 years	0
More than 10 years	5
**Specialist training**	
Care of older persons	1
Public Health	2
Midwifery	1
None	15

### Analysis

A qualitative content analysis with an inductive approach according to Elo and Kyngäs (Elo & Kyngäs, 2008), was used to analyse the data from the research persons of open-ended questions. The analysis consisted of three phases, and in the preparatory phase the data was read repeatedly to create an overall perspective and gain a deeper understanding of the content. Thereafter, in the organization phase, meaningful parts of the data, i.e., RNs free text responses on survey questions, were identified and the headings were formulated through an open coding. The headings from open coding describing the same thing on at an overall level were merged to create subcategories and categories. Discussions between the authors were conducted throughout the analysis process, as there was a risk that both the data collection and the results reflected the authors’ own preconceptions based on clinical experience on the subject. In the final phase, the reporting phase, the results were presented in two identified main categories with eight subcategories. An example of the implementation of the content analysis is shown in Appendix 2.

### Ethics

Approval for the study was obtained through the ethical board at Mälardalen University, Sweden, Dnr 2023/2927. When entering the Web link the RNs found an information letter with an informed consent page to print out and send back to the researchers. This procedure aimed to ensure that the RNs received relevant information about the study, understanding that participation was voluntary, and that consent to participate was obtained before they answered the survey. They were also informed that the collected information would be handled in a way that ensured only authorized persons would have access to it. Additional ethical aspects were considered in line with the Declaration of the World Medical Association in Helsinki (World Medical Association, 2014) and the Swedish Data Protection Agency GDPR (General Data Protection Regulation)

## Results

**Figure 1.**
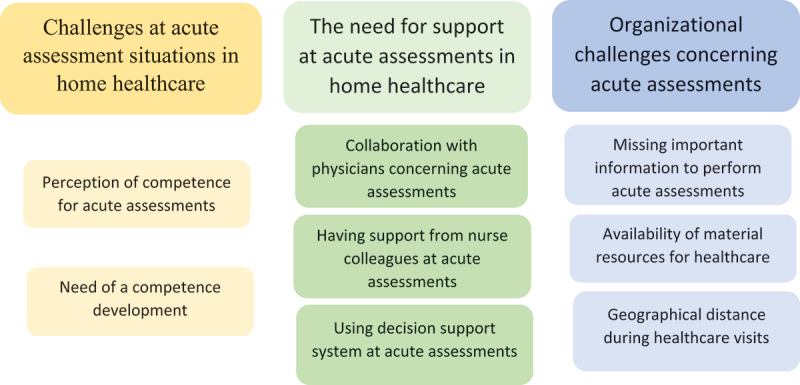
Categories and subcategories that emerged in the analysis and that show RNs’ experiences of acute assessments in the home.

The analysis resulted in three main categories: “Challenges at acute assessments in home healthcare”, “The need for support at acute assessments in home healthcare”, and “Organizational challenges concerning acute assessments” with eight subcategories. These categories and subcategories are presented according to the Figure 1.

### Challenges at acute assessment situations in home healthcare

The analysis showed that lack of competence was caused by a lack of experience with acute assessments in the home and because several of the RNs did not have much experience working as an RN and/or experience in home healthcare. The RNs also expressed a desire for more training in acute assessments.

#### Perception of competence for acute assessments

It emerged in the analysis that most of the RNs described that they had sufficient competence for patient-safe assessments, which was described by the nurses as carried out with relevant equipment by following assessment and treatment guidelines. Some RNs stated that they had the competence but limited experience in terms of clinical assessment and examination of patients who need an urgent assessment in home healthcare.

I feel that I have the skills to make assessments but still lack a lot of experience. (P 6)

The RNs who had longer experience in the profession claimed to be more confident based on their competence in performing acute assessments than those with less experience. Several RNs considered that it takes years of experience to possess the skills needed to perform patient-safe assessments.

I have worked for 3 years at an emergency department with a lot of triages in my work, so I think it has prepared me well. (P 5)

According to the analysis, it was common to have uncertainty due to limited experience with acute assessments and that experience was important for creating patient-safe and secure assessments in the home. RNs described that it takes years of experience to experience a sense of security at acute assessments in home health care.

I feel more confident in performing acute assessments now after having worked for a few years as an RN in home healthcare as I have gained more experience in that task. (P 1)

The RNs also described that experience gave a better clinical view for the acute assessments and that the uncertainty while performing assessments decreased over the years.

In addition to checking vital parameters, it is also about having a “clinical look” when meeting a patient to perform an assessment. A clinical look that you acquire over the years. (P 16)

#### Need of competence development

Several RNs expressed a desire for more competence development regarding acute assessments both for them as RNs in home healthcare and for the nursing staff in home care (i.e., assistant nurses and care assistants) regarding acute assessments, use of relevant equipment and emergency care. According to the analysis, it was not certain that nursing staff who contacted the RN have taken vital parameters or even have knowledge of how vital parameters are taken.

Better training for the nursing staff about symptoms and vital parameters and how to check them, also equipment for them that they bring with them at every visit, is needed”. (P 18)

More training for nursing staff in assessing what is urgent for a RN to assess on-call time and what is not needed. (P 5)

Due to the lack of competence and experience among nursing staff, it was difficult to determine the urgency of the condition of the patient who needed an acute assessment.

### The need for support at acute assessments in home healthcare

The analysis showed that in home healthcare, where the RNs often are unassisted during complex assessments, it is important to have support. Support from physicians, colleagues and DSS were considered to be important factors that could facilitate acute assessments.

#### Collaboration with physicians concerning acute assessments

According to the analysis, most of the RNs stated that the greatest challenge at acute assessments in home healthcare, compared to a health centre or hospital, was the limited availability of physicians.

The difficult thing is that you do not have access to a physician in the same way as in a hospital or health care centre, and that you are more alone and vulnerable in certain situations. (P 8)

Access to physicians was usually only available by telephone and only planned home visits were made together with physicians from the health care centre.

It can be difficult to find a physician within a reasonable time if you are unsure and need support and advice. (P 16)

Most RNs still considered that they had received good support from physicians when needed.

The RNs in the current study, preferred to have contact with physicians at the health care centre as those physicians had previous knowledge concerning the patients, while an on-call physician could only give advice based on what the RNs reported. Getting hold of a physician at the health care centre was described by some RNs as difficult as the call always had to go through an RN at the health care centre due to the organizational routines. The RNs wanted to always have a direct number for a physician who knows about the patient. The analysis showed that on weekdays it was usually quite easy to get hold of a physician and even easier to get hold of an on-call one. However, the quality of the call was not always perceived as good as it varied depending on the on-call physician.

During the day, our ordinary physician in home healthcare is worth his weight in gold! We have a physician who has retired but still works with home healthcare. We can always contact him on weekdays during normal working hours for consultation or assessment of our registered patients. At evenings and weekends, we contact the district on-call physician and the response you get can be very varied. But overall, it’s quite easy to get hold of a physician, which is good. (P 14)

### Having support from nurse colleagues at acute assessments

It turned out from the analysis that support from another RN provided support at acute assessments. The knowledge that there was a colleague to consult with gave the RNs a feeling that they were not completely alone in the situation. According to the RNs, it was a challenge to work in home healthcare because the work is lonely. They described that in a residence or hospital, there is a colleague usually in the next room who could support complex assessments or if assistance was needed. In home healthcare, the RNs were often alone in all decisions, and it could be difficult when experience was lacking. With a high workload when several assessments needed to be made, it could sometimes be difficult to prioritize which visit was most important and which needed to be made first. On these occasions, the RNs experienced that the support of a colleague would have been needed.

Almost never happens (is the only responsible RN on duty) as there is always another RN to contact by phone, but if I were all by myself there would be some concern. (P 2)

Yes, in the evenings we work alone, and it makes me feel bad sometimes. (P 13)

It emerged in the analysis that the possibility of contacting colleagues gave increased security as the colleagues could complement each other in an emergency situation. However, colleagues’ availability was a concern. Loneliness could be experienced both when the RNs had to assess unclear symptoms or when they lacked the presence of colleagues. Being alone in at assessment and not having access to any colleagues was a contributing factor to insecurity during emergency assessments at home.

It takes years of experience to become confident in making acute assessments in home healthcare. If I am unsure about something, I always call a colleague or a physician for advice. (P 16)

### Using a decision support system at acute assessments

All RNs in the study described that they work at home healthcare units where DSS must be used according to working routines and guidelines. Some of the RNs got the idea that the DSS could sometimes give a false sense of security in the assessment and there was a risk of staring blindly at what was on the paper instead of trusting their clinical assessment. However, the DSS was perceived to be a good support in the acute care and contributed to a structure in assessment and reporting about the patients’ symptoms and vital parameters.

However, some of the more experienced RNs considered that the DSS they use at their unit, was time-consuming and unnecessary, although it might be important for the RNs who were unsure and lacked experience.

A good instrument for those who are unsure but for me it feels a bit unnecessary, I don’t always use it I have to admit, even if I should”. (P 2)

DSS is good for the less experienced RNs and provides security in not missing anything during assessments. (P 3)

The less experienced RNs appreciated the DSS, and it gave increased security during an acute assessment if they were unsure, even as a support in case of uncertainty in connection with over-reporting to another healthcare provider.

Sometimes I feel completely safe, but often I am stressed and afraid to forget something in my assessment. We work with DSS, which makes me feel safer and it makes contact with physicians and SOS easier. (P 13)

### Organizational challenges concerning acute assessments

The analysis showed that in home healthcare, missing important information and limited access to equipment and materials are common organizational challenges. Since access to medical records was often insufficient, the RNs often lacked important information about the patients, which led to the assessments becoming more difficult to make.

### Missing important information to perform acute assessments

According to the analysis, several RNs expressed that they wanted access to medical records during the home visits as it was sometimes difficult to have time to read the medical record beforehand via computer in the office. Some of the RNs in the study had access to laptops or medical records via mobile phones, but still stated that they had limited access to medical records at home because the technology did not work as desired, e.g., no connection to internet.

We recently got a laptop that we can take with us, but it works only to some extent. (P 2)

It turned out from analysis that it was common not to have time to read the medical records beforehand, which made the assessment more complex and meant RNs were unable to answer any questions while over-reporting to the ambulance service. When RNs had time to read the medical record beforehand, they felt better prepared for the acute assessments.

Read through the medical record to get an idea of previous illnesses and medications. It is otherwise difficult to make a sensible assessment. (P 5)

When an acute assessment of an unknown patient needed to be made without first reading the medical record, the RNs stated that they tried to get help from a colleague to check important information if someone was available at the office or had a laptop with them, as well as through relatives or nursing staff if the patient could not answer for him/herself. By reading the medication list, which in most cases could be found in the home, the RNs could guess what types of diagnoses the patient might have had in the past and thus receive some support in their assessment.

If I haven’t had time to read the medical record and write down the most important things, I get as much information as I can from nursing staff and relatives, and check the medication list at the patient’s home, because prescribed medication can give me a hint about what type of patient it is. (P 4)

### Availability of material resources for healthcare

Not having time to pack the emergency bag or to check that everything was in it was described as often occurring by several RNs in the study. The RNs in the study explained that on the units there were shared emergency bags that they were responsible for refilling after use. They also mentioned that they had experienced occasions when something was missing from the emergency bag once they arrived at the patient’s home, which then led to an assessment being difficult to make. Therefore, they preferred having their own emergency bag with all the accessories needed for an assessment.

I would have liked each RN on duty to have her own bag with all the material needed for emergency assessments, so that each RN also has to take responsibility that everything is in the bag and works. (P 1)

The RNs also stated other complicating factors connected to performing assessments at home such as limited possibilities of taking samples and performing extended examinations such as electrocardiography. This made assessments difficult.

There is no physician nearby. Most of the time we are on our own. Often, we are far out in the forest in small houses, where there are no materials or equipment. That we have so few aids to control various things in the home. Hospitals and health centres have more equipment. (P 12)

### Geographical distance during healthcare visits

In the analysis, it emerged that according to the RNs, an acute assessment was usually initiated by telephone. The RNs stated that to be able to prioritize and assess what should be done, the information needed to be relevant and correct. It became even more challenging for RNs on-call because they were responsible for many more patients about whom they did not always have previous information about, and the geographical area was large, with a range of 30 kilometres from the office.

The difficulty is that it is sometimes far from the patient, usually working alone. An RN working nightshift and having sole responsibility for all enrolled patients. (P 4)

The RNs considered that transport caused a certain limitation in their assessment because it often took a long time and therefore it was difficult to monitor the patients, stay with them, or follow up on their status.

You don’t always dare to leave a deteriorating patient alone to come back after a while, if the patient had been in hospital, you could have waited and checked on him often, which is not possible if the patient lives alone in a small house in the forest. (P 12)

## Discussion

In this study, we examined Swedish RNs’ experiences of acute assessments at home. The results showed that the RNs considered themselves having the right skills and the opportunity to be prepared for acute assessments when working at home healthcare. They experienced that collegial support and the opportunity to consult physicians were important. Further, most of the RNs considered themselves as having good competence and possessing the competence needed for acute assessments, although they lacked experience. Previous research highlights that knowledge, clinical skills and broad medical competence are three important characteristics of RNs working with acute assessments in home healthcare (Andersson et al., 2017; Landstad et al., 2021). Competence among these RNs includes both theoretical and practical knowledge as well as experience, which is seen as an important part of the competence (Andersson et al., 2017). In the current study, 14 of the 19 RNs had less than five years of experience in home healthcare and only three had relevant specialist training. This can be assumed to be reflected in a perceived lack of experience that was expressed by several RNs. Research on pre-hospital assessments shows that the ability to value and organize the information that the RN is presented with during an acute assessment is more developed in the more experienced RN and that experienced RNs often base their assessment on previous experiences, which makes the assessment more secure (Andersson Hagiwara et al., 2014). Also, experience does not refer to age or how long one has held a position, but rather it is a process where one has developed one´s skills by being faced with real situations with patients in need of acute assessments. In addition, even experienced RNs may feel unsafe in situations they have not been in before (Rusli et al., 2022). This can also be related to patient safety, which in previous research by Landstad et al. (2021) has been shown to be strengthened if RNs have broad medical knowledge, i.e., work systematically, diagnose and manage specific medical conditions, and a good clinical competence as well as the ability to have a holistic view. RNs in home health care are also expected to possess a both broader and more advanced nursing competence (Narayan et al., 2017).

It emerged in the results that higher competence and knowledge was also needed among the nursing staff, and several RNs wished for more continuous competence development regarding acute healthcare and acute assessments both for themselves and for the nursing staff. This is also consistent with previous research (Bing-Jonsson et al., 2016). According to the RNs in the current study, it was not at all certain that the nursing staff who first visited the patient, measured vital parameters, or even had knowledge of how to measure. Lack of competence among the nursing staff is also common and can affect patient safety and make the RNs’ work more complicated (Bing-Jonsson et al., 2016). Also, lack of understanding and use of home healthcare competency scale could also hinder RNs’ professional or competency development (Rusli et al., 2023). With competent nursing staff who have patient knowledge, the RN can get good help during an emergency assessment. Delegating certain tasks to nursing staff is considered difficult as it is not possible to trust that they have the competence needed for the task (Bing-Jonsson et al., 2016). However, the fact is that the RNs in home healthcare are dependent on the nursing staff as the number of patients can be high and the distances to patients can be long. To prioritize and assess which patient is most in need of assessment, nursing staff need to have knowledge and the opportunity to assist RNs with the correct information about the patient’s status. *broad competence*

The results show that the DSS provided good support in assessment situations for the less experienced RNs, and they considered the use of the DSS to be very reassuring. The DSS provided structure and clarity in the assessment and support in reporting the patients’ status for the less experienced RNs, while those with more experience considered the DSS unnecessary and time-consuming. Previous research shows that experienced RNs more often use person-centred methods, i.e., focusing care on the needs of individual, during the thought process but also rely on their experience and intuition in a different way than those who are less experienced (Banning, 2008). Previous research also shows that in order to increase adherence to using the available DSS, they need to be adapted to the context in which they are used, e.g., home healthcare (Andersson Hagiwara et al., 2014). In a previous study among Swedish RNs, it appears that a DSS adapted for home healthcare was seen as very beneficial and safe when used together with vital parameters and the patient’s situation in general (Kihlgren et al., 2016). The results in the current study suggest that DSS can provide security and guidance in case of uncertainty in emergency assessments, although more experienced RNs believed that their own experience sometimes weighed heavier than a “red result” according to DSS. This may reflect a need for DSS to be further developed to fulfil its function.

The RNs in this study experienced an assessment more challenging if the patient was unknown and that the assessment could be flawed if there was no time to read the medical record, and it was also difficult to report to other care providers. This is also consistent with previous research, on situations where RNs do not have access to the patient’s medical record, with a lack of patient safety as a result (Bing-Jonsson et al., 2016; Dowding et al., 2020; Gray et al., 2018; Kihlgren et al., 2016). This is difficult to remedy in all situations, even if the technology were to develop due to no connection to the Internet, for example in rural areas. In addition, the RNs in the current study lacked correct medical technical equipment for patients with complex care needs. This is also highlighted in previous research showing that lack of correct equipment makes ensuring patient-safe assessments at home difficult (Berland et al., 2012; Bing-Jonsson et al., 2016; Gray et al., 2018; Landstad et al., 2021; Lindblad et al., 2018). RNs also described their work as lonely, and that loneliness and vulnerability can be experienced due to several factors such as not having any colleagues available when the assessment is challenging or if the patient lives far away. According to previous research, it is important to have support when working alone, both DSS and someone you can call to consult (Kihlgren et al., 2016). A more comprehensive collaboration with physicians also strengthens the patient’s safety and is a support for RNs in acute assessments in home healthcare (Landstad et al., 2021), as well as advanced nurse practitioners (Htay & Whitehead, 2021), a professional group that does not yet exist in primary care in Sweden. This is in line with the results from the current study, the RNs experienced the most difficult challenge in not having access to physicians in the same way as in inpatient care and primary care. In addition to a lack of support, it appears in Kaihlanen et al. (2023) study that many demands, such as an increased number of seriously ill patients, affect home care RNs’ well-being and job satisfaction with increased risk of burnout at the same time as a serious nursing shortage (Kaihlanen et al., 2023)., Our study shows that RNs often lack various aspects of support in their work and there is an urgent need to develop strategies to ensure sufficient personnel in home healthcare. An acute assessment at home carried out by an RN in home healthcare can be compared to a pre-hospital assessment carried out by RNs from the ambulance service. The difference is that the RN in home healthcare makes the assessment on her own without the support of a colleague and without the more advanced equipment that the ambulance service has.

### Strengths and limitations

We used a qualitative questionnaire to facilitate the recruitment of participants as a survey is faster to answer than an interview. The advantage with interview would have been that follow-up questions could have been asked, which was not possible with a survey. However, answers to questions of a more sensitive nature may be easier to get via a questionnaire where anonymity is guaranteed. The pilot survey increased the study’s quality and credibility as it gave us information about the questions seemed to respond to the purpose of the study. Validity and reliability are acceptable as we asked for the RNs’ own experiences and because the answers cannot be linked to a specific individual. A purposive sample was applied to obtain answers for the purpose of investigating RNs’ experiences of acute assessments in the home. At first it was difficult to get enough responses. It is unclear if the response rate was low related to the RN’s high workload or if emails got lost in the email flow. A common negative aspect regarding web surveys via email is that emails that are supposed to be answered later are forgotten. We also obtained a wide selection of participants both in terms of age and number of years in the profession, which in turn strengthens the credibility and generalizability of the results. Although the number of years in the profession was less than five for most RNs, there was a range and, in the results, this appears as less experienced or more experienced RNs. All RNs employed on the units were invited, but it can be seen as a limitation that no men consented to the study. In general, very few male RNs work in this context, so no special measures were taken to include male participants. An inductive content analysis was thought to be a suitable method as it is a flexible method. A limitation is that the analysis was carried out by the first author, however, the process was strengthened through discussions with the other authors.

It can be seen as a hindrance that the authors have worked in the study field as it can increase pre-understanding. On the other hand, throughout the course of the analysis, awareness of pre-understanding and the risk of influence has been present, and the results have been discussed between the authors. A certain pre-understanding can be beneficial for the study as the subject is familiar to the authors and credibility can also be strengthened by the researchers being familiar with the subject.

## Conclusion

The knowledge from this study contributes to an increased understanding of RNs’ experiences of acute assessments within home healthcare. Acute assessments are complex due to several factors. To facilitate acute assessments at home, RNs need to have the opportunity to access the patient’s medical record at home, have collegial support and have the availability to contact physicians in connection to an acute assessment. For evidence-based assessments to be made on equal terms, more competence and adapted user-friendly DSS are needed in home healthcare. More competence development is also desired for the nursing staff because acute assessments in the home are usually preceded by a call from the home care service. The results show also that collaboration between primary healthcare, on-call physicians and nursing staff is important and having the opportunity to consult with someone provides security in acute assessments.

In terms of clinical implications, the current study shows the importance of implementing a decision support that even more experienced RNs finds benefit in using. Furthermore, the importance of further investigating which training needs is current for the home care staff and developing decision support adapted for the nursing staff in the home care to further increase patient safety.

## Data Availability

The datasets generated and/or analysed during the current study are not publicly available due to Swedish regulations and laws but are available from the corresponding author on reasonable request.

## Geolocation information

The study was conducted in western Sweden, in northern Europe.

## Notes

Appendices

## Appendix 1. Survey questions used in current study to explore Swedish registered nurses’ experiences of acute assessments in the home

**Table:**

1. Are you male or female?
2. How old are you?
3. How long have you worked as a nurse?
4. How long have you worked as a nurse in home health care?
5. If you have any additional training in addition to basic training as a nurse, state which one(s):
6. You receive a call about an urgent assessment. Describe how you prepare and what you feel and think about going for an assessment.
7. Does it happen that you are the only responsible nurse on duty and what are your feelings about that?
8. How would you describe your ability and competence in making acute assessments?
9. During home assessments, it is still common for RNs not to have access to patient records and other information about the patient at home. Can you tell us about what it looks like in your unit and how you solve the challenge of not always having access to all the necessary information on site in the patient’s home?
10. What difficulties and challenges do you think there are in acute assessments at home compared to, for example, a hospital or health centre?
11. What circumstances could contribute to you feeling that the assessment feels more challenging? For example, any particular time of day, the patient’s condition or age?
12. Do you have any assessment support at your workplace to be used for urgent assessments? If so, describe which one and what you think about it. Ex. if it is always available, if it is used and if it provides good support in assessments.
13. Do you think that you get the support you need from physicians when you need advice and support during an assessment at home or is there anything you wish was different?
14. If you yourself had to choose some areas of improvement within acute assessments at home in your unit, what could your unit do to improve for you and your colleagues in situations where acute assessments need to be made?
15. Thank you for taking the time to participate in this survey. If you want, you can leave a voluntary comment below if you have anything to add in addition to your answers to the questions.

## Appendix 2. An example of the implementation of the content analysis

**Table:**

The preparation phase	The organization phase		The reporting phase	
*Registered nurses free text responses on survey questions*	*Headings from open coding*	*Merged headings describing the same thing on an overall level*	*Subcategory created from the headings*	*Category, an abstraction of the content*
I find it exciting to go out for assessments in the home, I feel that the staff gives some of the information, the patient some, relatives some, the medical record some and my experience some. I am not afraid of making the wrong assessment because when in doubt I always have the option of calling the physician for telephone advice.	Not afraid to make mistakes, can always call a physician for advice	Confident in the role as RN	Perception of competence for acute assessments	Challenges at acute assessment situations in home healthcare
If I’m out in the car, I sometimes call a colleague who might be at the computer to get information, otherwise I go to the pat and take it from there.	Calls a colleague and asks for information on a home visit	Safe with a colleague	Having support from nurse colleagues at acute assessments	The need for support at acute assessments in home healthcare
